# The dark-ventral-patch of male red deer, a sexual signal that conveys the degree of involvement in rutting behavior

**DOI:** 10.1186/s40850-021-00083-9

**Published:** 2021-05-28

**Authors:** Eva de la Peña, Javier Pérez-González, José Martín, Giovanni Vedel, Juan Carranza

**Affiliations:** 1 Wildlife Research Unit (UIRCP), University of Córdoba, 14071 Córdoba, Spain; 2 Biology & Ethology, University of Extremadura, 10071 Cáceres, Spain; 3 Department of Evolutionary Ecology, Museo Nacional de Ciencias Naturales (MNCN-CSIC), 28006 Madrid, Spain

**Keywords:** Red deer, Dark ventral patch expression, Mating effort, Sexual behaviors, Mate competition, Mating success

## Abstract

**Background:**

In polygynous mammals, signalling may play a decisive role in mating behavior, mediating the intensity of male fights and female mate choice. During the rutting season, male red deer may show a visible dark patch in their ventral fur. Recently, this patch has been suggested to act as a flexible sexual signal, due to its relationships with other variables such as age, body size, antler development, volatile compounds, or the competitive environment. The analysis of fur pigmentation at the ventral patch suggests that this might also visually indicate the male intrinsic predisposition to take part in mating competition.

**Results:**

To assess the possible role of this trait as a communicative signal related to mate competition, we used red deer behavioral observations during the rut in Doñana National Park (Spain) to examine the link between the degree of expression of the dark ventral patch and the rutting activity (assessed from both intra-and-inter-sexual behaviors). Consistent with our predictions, we found in a field study that males with large dark patches showed a higher frequency of rutting behaviors (mainly roaring and flehmen), more interactions with females, and attained larger harem sizes.

**Conclusions:**

The dark ventral patch was a better predictor of male behavior than antler tines or territory holding, thus standing as a short-term indicator of male willingness to invest in mating competition.

**Supplementary Information:**

The online version contains supplementary material available at 10.1186/s40850-021-00083-9.

## Background

Signalling is a widely studied aspect of animal behavior, where individuals, within or between species, interact [1, 2]. Signalling plays a key role in sexual selection [3]. Under sexual selection, mating success depends upon the intrasexual competition and the mating preferences of the opposite sex [3, 4], and both rivals and mates may use information from signals that honestly reveal quality [5–7].

Honest signalling has been proven to be highly relevant in the sexual selection processes of many species of vertebrates [8]. For example, brightly colored ornaments in birds (*Lagopus lagopus scotica* [9]; *Alectoris rufa* [10]; *Sula nebouxii* [11] and in reptiles (*Testudo hermanni* [12]; *Trachemys scripta elegans* [13]; *Iberolacerta cyreni* [14]) act as reliable signals of individual quality. The variation in black plumage traits in birds also plays a key role during the intrasexual competition, revealing male dominance rank (e.g., the black bib in *Carduelis spinus* [15]; the badge in *Passer domesticus* [16–18]).

In polygynous species, male success in intrasexual competition is a decisive factor on fitness, and males develop weapons and ornaments to face rivals and attract potential mates [3]. Male competition over access to females is often resolved by means of direct intrasexual agonistic interactions resulting in aggressive contests [19]. However, there are signals acting alongside non-injurious behaviors in mate competition that play an important role in intrasexual communication [20]. Furthermore, there are signals that encompass information about both an individual’s quality and on its behavioral strategy, in many cases related to reproduction [15, 21, 22]. Alternative strategies may involve some phenotype variables such as age or dominance rank but also environmental factors, such as the mate competition context [23].

In male red deer (*Cervus elaphus*), there is a large repertoire of behaviors that occur during the mating season aimed at maximizing the number of females to mate with. While gathering female harems or mating territories, stags roar repeatedly [24–26], and this behavior is positively associated with both reproductive success and fighting ability [24, 26], but also depends on their body condition [27]. Agonistics encounters between males can be frequent, fights being the most conspicuous of these behaviors. As a result of the fighting, around one-fifth of rutting males can show signs of injury [24]. Prior to fighting, males repeat a sequence of behaviors with which they can assess the competitive ability of their opponents [24]: both males stand in a visible position several meters apart, roaring towards the opponent. After some minutes exchanging roars, a second stage begins in which the challenger moves to meet his rival. When challengers are separated a few meters apart, they initiate a parallel walk, usually at right angles to the direction they approached. After the parallel walk, a fight can start or be avoided [24].

In red deer, antler size is a sexual character positively related to winning contests and mating success [28, 29]. On the other hand, roaring is a good signal of body size, with lower formant frequencies being related to reproductive success [30]. Roaring has been considered an index of fighting ability since only big males can produce roars with lower formant frequencies [31, 32]. Recently, the dark ventral patch has been described as a complementary sexual trait in red deer [33–37]. This dark patch has a discrete, bimodal distribution in size [38] that might play a relevant role in communication during the rut. The hairs of this ventral area are impregnated by strongly odoriferous compounds that are related to some male characteristics such as age, and intrasexual competition level in the population [33, 35]. These compounds might inform about the genetic identity and quality of a male as well as about variables such as age or body condition. Furthermore, recent studies have shown that the investment in volatile compounds in the dark ventral patch is also dependent on the social competitive environment; individuals under high mate competition make a greater investment in volatile compounds than those under low mate competition [35]. Moreover, the analysis of fur pigmentation at the dark ventral patch suggests that this trait might also visually indicate males’ fighting capacity [34]. Therefore, the size of the ventral path in male red deer might be used as a communicative signal related to rutting activity. In this case, signalling individual fighting ability by the dark ventral patch may alter the behavior of other males during rival assessment, thereby increasing their fitness, in a system where both parties benefit [8, 32].

The expression of the dark ventral patch in male red deer would be a communicative signal if it is related to changes in the behavior and fitness of both the sender and the receiver. Here, we assessed the possible role of this trait as a communicative signal [34, 38] from the sender’s perspective. Accordingly, we hypothesized that the dark ventral patch in male Iberian red deer (*Cervus elaphus hispanicus*) is related to rutting behaviour and mating success. We predicted a positive relationship between the expression (size) of the dark ventral patch of a male and (1) his rutting activity, and (2) the number of females gathered. For this assessment, we took also into account other individual characteristics such as age category, territoriality, and number of antler tines. The use of different individual features, in addition to the dark ventral patch, allowed the comparison of their relative effect on rutting behavior.

## Results

Table 1 summarizes the number of individuals corresponding to each level of ventral patch expression, territoriality, and age categories. Throughout the 4 mating seasons, the number of territorial males (*N* = 8) for which behavioral data were taken was lower than the number of non-territoriality individuals (*N* = 64). Similarly, behavioral data were recorded mostly from adults (*N* = 60) rather than subadult males (*N* = 12).

**Table 1:** Summary of data and classifications of sampled individuals corresponding to each category of territoriality (territoriality vs. non-territoriality) and age (subadult and adult) by year of male Iberian red deer (*Cervus elaphus hispanicus*)

		*Subadult*		*Adult*	
*Year*	*Trait expression*	*Territoriality*	*Non-Territoriality*	*Territoriality*	*Non-Territoriality*
2015	LTE	0	1	0	0
	HTE	0	0	3	1
2016	LTE	0	2	0	2
	HTE	0	0	3	3
2017	LTE	0	9	0	8
	HTE	0	0	2	21
2018	LTE	0	0	0	0
	HTE	0	0	0	17

The mean frequency of sexual behaviors for males of both levels of trait expression and the comparisons between them are presented in Table 2. Sexual behaviours in subadult or younger males were very scarcely recorded. For adult males, rates of roaring, female harassment and sexual activity were significantly higher for HTE males compared to LTE males. The remaining behaviors did not show significant differences in mean rates between both groups of males. It is worth highlighting that despite the dark ventral patch is a flexible trait, all observed males in this work remained with the same trait score during the rutting season. Similar results (Additional file 1: Appendix S1) were found with the subset data from 2016 and 2017, which includes adult males from both groups of trait expression level (LTE and HTE; see Table 1).

**Table 2:** Frequencies (behavior rate per minute) of male Iberian red deer (*Cervus elaphus hispanicus*) mating behaviors displayed during four consecutives rutting seasons of both adults (*N* = 209 observations; *N* = 322 recorded behaviors) and subadult males (*N* = 18 observations; *N* = 8 recorded behaviors). In case of adults, table also shows mean and standard deviations of frequencies, as well as Generalized Mixed Models results in which the frequency of each behavior was included as the dependent variable, the level of trait expressions (LTE vs HTE) as fixed factor, and both individual (*N* = 60) and year (2015, 2016, 2017, 2018) as random factor. Each line in the GLMMs columns shows the mean differences and standard errors (SE) between trait expression, the Z statistic and significance level. The number of observations (Obs) in which a particular behaviour was recorded are also shown in brackets

Mating behaviour	Mean frequency ± SD		GLMMs		
	LTE	HTE	Estimate ± SE	*Z*	*P*
**Roaring**					
Adults (Obs = 141)	0.83 ± 0.90	2.13 ± 1.52	−2.05 ± 0.98	−2.10	0.036
Subadults (Obs = 5)	–	–	–		–
**Flehmen**					
Adults (Obs = 38)	0.01 ± 0.05	0.13 ± 0.41	−6.78 ± 7.99	−0.84	0.40
Subadults (Obs = 0)	–	–	–	–	–
**Antler rubbing**					
Adults (Obs = 49)	0.08 ± 0.26	0.14 ± 0.37	0.01 ± 0.31	− 0.09	0.92
Subadults (Obs = 1)	–	–	–	–	–
**Parallel walk**					
Adults (Obs = 10)	0.01 ± 0.05	0.03 ± 0.13	−1.06 ± 2.01	−0.53	0.59
Subadults (Obs = 0)	–	–	–	–	–
**Fight**					
Adults (Obs = 4)	0.03 ± 0.11	0.01 ± 0.04	0.57 ± 0.31	1.85	0.06
Subadults (Obs = 0)	–	–	–	–	–
**Female harassment**					
Adults (Obs = 74)	0.05 ± 0.15	0.31 ± 0.48	−5.27 ± 4.55	−1.58	0.24
Subadults (Obs = 2)	–	–	–	–	–
**Mount female**					
Adults (Obs = 6)	0.00 ± 0.00	0.01 ± 0.05	− 25.39 ± 377.1	−0.07	0.94
Subadults (Obs = 0)	–	–	–	–	–
**Sexual activity**					
Adults (Obs = 209)	1.02 ± 0.94	2.76 ± 1.93	−2.05 ± 1.02	−2.02	0.04
Subadults (Obs = 18)	–	–	–	–	–

The following models were conducted for sexually mature (adult) males (see Methods). For sexual activity (summation of of all seven sexual behaviors, see Methods), males with high ventral dark patch expression (HTE) were more sexually active than LTE ones, after controlling for the number of antler tines, harem size and territoriality (territorial vs. non-territorial) (LMM1 in Table 3). Interestingly, antler tines, harem size, and territoriality did not show any significant effect on sexual activity when dark patch expression was in the model.

**Table 3:** Results of LMM1 for the effect of ventral patch expression (LTE vs HTE) of adult male Iberian red deer (*Cervus elaphus hispanicus*) on male ‘sexual activity’ for which there were data for both LTE and HTE males. Analyses controlled for the number of antler tines and territorial behaviour. Reference levels for factors are shown in brackets. Significant effects are showed in bold (*p*-value = 0.05). Table also shows variance and standard errors (SE) of random effects (individual and year), as well as the residual variance of the model

Dependent variable: Sexual activity	Estimate (± SE)	df	*T*	*P*
Fixed factors				
Intercept	3.387 ± 0.805	17.077	4.209	**0.002**
Trait expression (HTE)	- 1.760 ± 0.518	188.123	−3.397	**0.001**
Antler tines	- 0.059 ± 0.063	40.652	−0.952	0.392
Territoriality (Non-territoriality)	0.294 ± 0.388	15.101	0.758	0.504
Harem size	0.011 ± 0.043	163.619	0.249	0.811

Random factors:

*Individual*: variance ± SE = 0.000 ± 0.000; *Year* = 0.037 ± 0.189; Residual = 3.546 ± 1.883

When exploring the factors affecting mating success, as approximated by harem size, we found significant effects of both dark ventral patch expression and number of antler tines, independently of whether males were territorial or not, or their sexual activity (Table 4).

**Table 4:** Results of GLMM fitted to a Poisson distribution for the effect of ventral patch expression (LTE vs HTE) of adult male Iberian red deer (*Cervus elaphus hispanicus*) on male harem size for which there were data for both LTE and HTE males. Analyses controlled for the number of antler tines, territoriality (territoriality vs non-territoriality), and male sexual activity. Reference levels for factors are shown in brackets. Significant effects are showed in bold (*p*-value = 0.05). Table also shows variance and standard errors (SE) of random effects (individual and year)

Dependent variable: Harem size	Estimate (± SE)	Wald Chi-square	*P*
Fixed factors			
Intercept	- 0.367 ± 0.520	0.077	0.781
Trait expression (HTE)	- 0.355 ± 0.213	7.902	**0.005**
Antler tines	0.078 ± 0.032	5.872	**0.015**
Territoriality (Non-territoriality)	- 0.111 ± 0.107	1.076	0.299
Sexual activity	- 0.009 ± 0.159	2.581	0.108

Random factors:

*Individual*: variance ± SE = 0.248 ± 0.498; *Year* = 0.000 ± 0.000

The PCA for the seven defined mating behaviors (Table 5) of adult males along the four sampled rutting seasons produced four principal components (PCs) with eigenvalues greater than 1 (Table 6). The first component (PC1) explained a 20% of the variance. It was characterized by a high contribution of roaring and flehmen, and to a lesser extent by antlers rubbing, female harassment, and parallel walk. All the correlations between the variables and this feature axis PC1 were positive. Hence, the first principal component PC1 encompassed most of the rutting activity. PC2 accounted for 19.9% of the variance and it was positively correlated with female harassment and flehmen and negatively with antler rubbing. The PC3 accounted for 14.8% of the variance and the main loading was for mating attempts (mounting). Finally, the PC4 explained the 14.4% of the variance and was contributed by the fighting behaviour.

**Table 5:** Reproductive behaviors of male Iberian red deer (*Cervus elaphus hispanicus*; *N* = 72) reproductive behaviors displayed during four consecutives rutting seasons (*N* = 227 observations; *N* = 330 recorded behaviors). The number of times a behaviour was recorded is shown as Obs. (observed)

	Description
Roaring (Obs. = 146)	Loud call that males repeat frequently during the rut.
Flehmen (Obs. = 38)	The male raises his head and stretches the neck forward, and with the mouth opened the upper lip was curled back.
Antler rubbing (Obs. = 50)	Rubbing of shrubs and trees where the male sways his head sideways, sometimes picking up branches and leaves that remain in the antlers.
Parallel walk (Obs. = 10)	Two stags walk side by side usually turning to face its opponent and lowering its antlers. During the walk males are often roaring.
Fight (Obs. = 4)	Males intertwine their antlers and struggle with the opponent by shaking their heads sideways, trying to push back the rival male.
Female harassment (Obs. = 76)	The male pushes his face into the female’s anogenital region and runs after her for more than 5 s.
Mount female (Obs. = 6)	Attempts to copulate, where the male rests his body on the back of the female.

**Table 6:** Principal components analysis for sexual behaviour variables during the rut in male Iberian red deer (*Cervus elaphus hispanicus*). Correlations between variables and the principal components greater than 0.40 are marked in bold. Eigenvalues proportion of variance and cumulative variance for each component are shown

	PC1	PC2	PC3	PC4
Roaring	**0.674**	−0.199	0.033	0.092
Flehmen	**0.439**	**0.516**	−0.041	−0.073
Antler rubbing	0.368	**−0.496**	0.106	−0.031
Parallel walk	0.275	−0.353	−0.355	− 0.346
Fight	0.042	−0.091	−0.395	**0.889**
Female harassment	0.367	**0.546**	0.037	0.058
Female mount	0.068	−0.121	**0.838**	0.267
Eigenvalue	1.451	1.399	1.036	1.001
Proportion of variance	0.207	0.199	0.148	0.144
Cumulative proportion	0.207	0.407	0.555	0.698

**Fig. 1:**
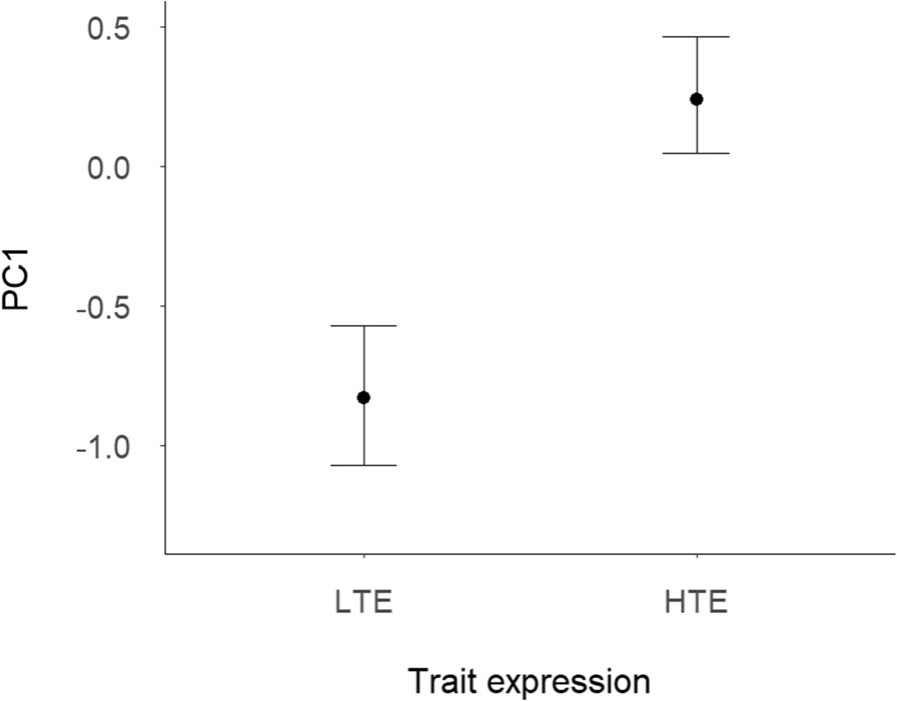
Sexual behaviour represented by PC1 relative to ventral patch expression (LTE vs HTE) on adult male Iberian red deer (*Cervus elaphus hispanicus*)

Derived from the LMM2 (i.e., PC1 as the response variable), we found a significant effect of the dark ventral patch expression after correcting for the number of antler tines (Table 7A). Thus, HTE males showed higher values of PC1 (i.e., higher roaring and flehmen rate, related to rutting activity) than LTE males (Fig. 1). However, in the case of LMM3, LMM4, and LMM5 (Table 7B, C, D) using the PC2, PC3, and PC4 as the dependent variables respectively, we did not find any significant effects of the dark ventral patch expression controlling for the number of antlers tines.

**Table 7:** Results of LMM2 (**A**), LMM3 (**B**), LMM4 (**C**) and LMM5 (**D**) for the effect of ventral patch expression (LTE vs HTE) of adult male Iberian red deer (*Cervus elaphus hispanicus*) on the Principal Component PC1 (**A**), PC2 (**B**), PC3 (**C**) and PC4 (**D**) representing reproductive activity for which there were data for both LTE and HTE males. Analyses controlled for the number of antler tines. Reference levels for factors are shown in brackets. Significant effects are showed in bold (*p*-value = 0.05). Table also shows variance and standard errors (SE) of random effects (individual and year), as well as the residual variance of the model

(A) Response variable: PC1	Estimate (± SE)	df	*t*-value	*p*-value
Fixed factors				
Intercept	- 0.343 ± 0.443	11.168	- 0.774	0.539
Trait expression (HTE)	0.507 ± 0.158	192.233	3.197	**< 0.002**
Antler tines	- 0.002 ± 0.032	24.066	- 0.068	0.953
Random factors: *Individual*: variance ± SE = 0.000 ± 0.000; *Year* = 0.009 ± 0.158; Residual = 1.369 ± 1.170				
**(B) Response variable: PC2**	Estimate (± SE)	df	*t*-value	*p*-value
Fixed factors				
Intercept	- 0.746 ± 0.623	20.458	- 1.198	0.299
Trait expression (HTE)	0.088 ± 0.168	187.892	0.526	0.605
Antler tines	0.043 ± 0.040	39.293	1.050	0.351
Random factors: *Individual*: variance ± SE = 0.064 ± 0.252; *Year* = 0.157 ± 0.396; Residual = 1411 ± 1188				
**(C) Response variable: PC3**	Estimate (± SE)	df	*t*-value	*p*-value
Fixed factors				
Intercept	- 1.404 ± 0.714	18.268	- 1.974	0.073
Trait expression (HTE)	- 0.129 ± 0.129	180.146	0.997	0.326
Antler tines	0.116 ± 0.041	68.564	2.850	**0.008**
Random factors: *Individual*: variance ± SE = 0.262 ± 0.512; *Year* = 0.596 ± 0.772; Residual = 0.614 ± 0.784				
**(D) Response variable: PC4**	Estimate (± SE)	df	*t*-value	*p*-value
Fixed factors				
Intercept	- 1.404 ± 0.711	18.268	- 1.974	0.074
Trait expression (HTE)	- 0.129 ± 0.129	180.146	0.997	0.326
Antler tines	0.116 ± 0.041	68.564	2.850	**0.008**
Random factors: *Individual*: variance ± SE = 0.262 ± 0.512; *Year* = 0.596 ± 0.772; Residual = 0.614 ± 0.784

When taking into account only the two years (2016 and 2017) in which we obtained data of adult males of the two types (LTE and HTE) and considering both years separately, no variable significantly affected the PC1 in the 2016 small data size subset (Additional file 1: Appendix S2 A), while for 2017, where the sample size was larger, the effect of the dark ventral patch expression on the PC1 was significant (Additional file 1: Appendix S2 B). Indeed, the Bonferroni correction showed an effect of the dark ventral patch expression on the PC1 (*P* = 0.025). These results were confirmed by using LMMs and bootstrapped significance for the effect of trait expression on the PC1 (Additional file 1: Appendix S3). The same result was found by calculating robust model estimates using data from the two years joined together (*P* <  0.001).

## Discussion

Our results support the hypothesis that adult male red deer with a higher expression of the dark ventral patch showed more sexual activity regarding the rate of some rutting behaviors (mostly roaring and flehmen) compared to low trait expression males. This was so even though both types of males were sexually mature, as shown by their participation to some extent in mating behaviors. Moreover, high trait expression was related to larger harem sizes. According to the sender’s point of view [32], our results show that the dark ventral patch of male red deer may act as a flexible sexual signal. This signal might indicate not only individual features such as condition or age [33–35], but also the degree of male involvement in rutting behavior and intrasexual competition during the current mating season. It is worth highlighting that the effect of the dark-ventral-patch expression on the sexual behavior found here was independent of other males’ attributes, such as the number of antler tines, whether the male was territorial or not and his harem size, thus supporting the role of the ventral patch as the main indicator of male sexual activity.

Previous studies have demonstrated that the expression of the dark ventral patch is the consequence of the deposition of volatile compounds on the fur [33]. These volatile and odoriferous compounds can be used as chemical information to communicate genetic features, age, or rutting involvement of individuals [33, 35]. Moreover, it has been shown that this trait may play a role as a visual signal in rutting interactions between males [34, 36, 37]. All these findings support the idea that dark ventral patch expression has a major signalling role during the rut.

Male red deer have weapons, the antlers, which can cause serious injuries during mate competition [24]. Due to aggressive interactions being energetically costly and potentially risky, mechanisms for assessing their opponents before the combat are expected to evolve in this species. These mechanisms might imply the use of reliable signals that inform about competitive ability or aggressive willingness [8]. Displaying behaviors such as parallel walk [24], roar acoustic characteristics [30], or chemical compounds [33] are important cues to assess challengers in our model species. Thus, we could assume that visual signals might play an important role in rival assessment. Interestingly, our results show that the expression of the dark ventral patch was more related to rutting activity than antler tines, territoriality, or harem size. This result agrees with the suggestion that the dark ventral patch expression provides more proximate information about individual attributes [38], thus allowing rival assessment to be most accurate at the moment when it takes place. Therefore, we suggest that the expression of the ventral patch might be the most important signal to evaluate, at least, the aggressive or competitive willingness of opponents.

The evolution of the dark ventral patch as a reliable visual signal may have been favored by social costs [39] in the red deer mating system. A young subordinate male might deceive rivals by producing an apparently little costly signal such as a big dark ventral patch. However, the potential benefits of this cheating behavior are not clear, while deceivers may suffer important injuries (i.e., social costs) by genuinely dominant males with high aggressive motivations [40]. Even if cheaters were able to attract females, they should be prepared to maintain the continuous challenge by other males that try themselves to achieve copulations [28, 41]. The possible high social costs of developing a high trait expression might explain why young, though sexually mature, individual males with large dark ventral patches are scarce. While acoustic and chemical signals may inform about features such as body size, condition, or age [30, 31, 33, 35, 42], the size of the dark ventral patch of male red deer might also inform about the current degree of male involvement in the mating competition. As a visual signal, the dark ventral patch might be considered as a handicap in which honesty would maintain by the social costs related to the risk of antagonistic interactions with strong dangerous challengers [32]. Thus, the dark ventral patch in red deer might be analogous to black patches in different bird species [15, 43]. A similar function as visual signal under the handicap context might be assigned to black patches found in mammal species such as white-tailed deer (*Odocoileous virginianus* [44, 45] and mule deer *(Odocoileous hemionus* [46]).

Signals that modulate the intensity of overt fighting are common in several species [47]. Visual signals may encompass information on dominance, territoriality, and fighting ability in both invertebrates (*Oreochromis niloticus* [48]; *Perisesarma eumolpe* and *P. indiarum* [49]) and vertebrates (in birds such as *Agelaius phoenicus* [50]; *Passer domesticus* [51]; *Euplectes ardens* [52]; see above; and in lizards such as *Lacerta agilis* [53]; *Psammodromus algirus* [40]; *Chlamydosaurus kingii* [54]; *Anolis sagrei* [55]; *Podarcis muralis* [56]). However, in contrast to birds or lizards, in mammals, there is much less evidence of chromatic signals. Nevertheless, in some primate species, it has been shown that colorful patches or the degree of the scrotal color of males predict dominance [57, 58]. Also, in lions, dark-maned males are preferred by females and are more likely to win in mate competition fights [59].

The dark ventral patch expression was positively related to the frequency of mainly both roars and flehmen. Stags usually roar as they gather harems of hinds [24–26] or while defending their territories against rival males during the rut [24, 60]. On the other hand, flehmen is a chemosensory response to females’ urination also during the rut [61, 62] and it is influenced by social structure at mating in several mammal’s populations (*Kobus defassa* [63]; *Capra hircus* [64]; *Lasiorhinus latifrons* [65]). Similar to what has been shown in the waterbuck (*Kobus defassa*) [63], red deer males usually mount females after displaying flehmen [66] and this behavior is typical of those individuals that maintain a harem.

Likewise, we found that the number of females in a harem was related to the ventral patch expression of a male. Variations in harem size are expected to relate to the variance in reproductive success among males [67] since the efforts to maintain and monopolize a group of females entail high costs that should be compensated by fitness benefits [68]. Pemberton et al. [69] showed that behavioral estimates of mating success were reliably related to actual male reproductive success.

We did not find a strong relationship between the dark ventral patch and fighting. Because of the risks of being injured, which can even lead to death [24], or the probability of losing females from the harem [28], stags try to avoid overt fighting [24] and hence escalated contests in the wild are scarce. Males with the largest ventral patches may be characterized by expressing sexual behavior and attaining larger harems but not by being involved in more fights than other males. Other behaviors such as antler rubbing and parallel walk also appeared not significantly related to the dark ventral patch, suggesting that they may not be signalling fighting ability. But also, sample size limitations may prevent significant results, as for instance for mounting attempts, for which we could expect a relationship with high trait expression.

This work presents limitations related to the sample. Firstly, the sample size is small, mainly due to the difficulty of taking behavioral data from large mammals in the wild, and because most reproductive behavior takes place at night in this species. In order to avoid invasive procedures to age-determination, visual methods have been used to classify males as young or adult. Furthermore, the impossibility of marking individuals along sampling years presents an additional limitation, which may lead to a pseudo-replication error between years. However, this study throws light on future experimental studies that modify the size of the dark ventral patch to reveal differences in behavioral response of both male and female deer during the rut.

Here we found evidence on the role of the dark ventral patch in male red deer as a visual signal from the sender’s point of view. Future studies might focus on the receiver’s perspective. Experimental approaches such as those manipulating the size of the visual signal [70–73] might be implemented in red deer populations. These experiments might help to increase the understanding of its action as a visual signal from the perspective of both the sender and the receiver.

The expression of the dark ventral patch might also be used in male-female interactions. Females may assess genetic features and the rutting involvement of potential mates [42, 74]. It is likely that the chemical compounds present in the dark ventral patch may provide information to females on male features [33, 35] but we do not know whether females use the visual character as a cue. An experimental approach may be necessary here to disentangle the visual role of the trait as well as the relative effects of intra- and intersexual selection components in the evolution of the dark ventral patch.

## Conclusions

In conclusion, our study for a red deer population in Southern Spain has revealed that the dark ventral patch expression may play a relevant role as a signal related to male sexual activity during the rutting season The dark ventral patch may act as a flexible sexual signal conveying information on male features (dominance and age) but also on his involvement in mating competition. These results suggest that the size of the dark ventral patch encodes two different behavioral tactics of male red deer when facing each rutting season.

## Methods

### Study area

Fieldwork was carried out during four consecutive years, between 2015 and 2018, in the Doñana Biological Reserve (RBD-CSIC) within the limits of the Doñana National Park (SW Iberian Peninsula). This is an area with a typically Mediterranean climate, with hot and dry summers and the rainy season concentrated during autumn and spring [75, 76].

We made observations of Iberian red deer behavior during their mating season, which in this area occurs during September [41], just after the season with the highest scarcity of food resources has finished, when deer gather in those meadows with some remaining pasture patches [77]. Mean rainfall in Doñana during the four sampled years was 482.8 mm, and the mean temperature was 19.4 °C. The lowest average annual temperature was registered in 2016 with 17.8 °C and, the highest average annual temperature was reached in 2018 with 24.8 °C (data collected from ICTS-RBD available at http://icts.ebd.csic.es/).

The study area is characterized by a seasonal marsh ecosystem, with marshy vegetation based on *Scirpus maritimus*, *S. littoralis* and *Phragmites australis*. Marsh is bounded by a characteristic dark and dense scrub called ‘monte negro’ (formed by *Erica scoparia, E. umbellata, E. ciliaris, Calluna vulgaris, Myrtus communis, Phillyrea angustifolia, Rubus ulmifolius, Ulex minor, U. australis).* The contact between marsh and shrub occurs along a strip of land called ‘vera’, an ecotone of open meadows and high ecological richness.

We made observations in an area centered around a deer mating place at the ‘vera’ habitat, from a raised structure (scaffolding) that allowed us to visually cover a total of 60 ha. In this area, the behavior of red deer during the rutting season has been extensively studied [41, 60, 78–81]. The number of different individual red deer (both males and females) observed in a year was 72 ± 11 (mean ± SE).

### Expression of the dark ventral patch

**Fig. 2:**
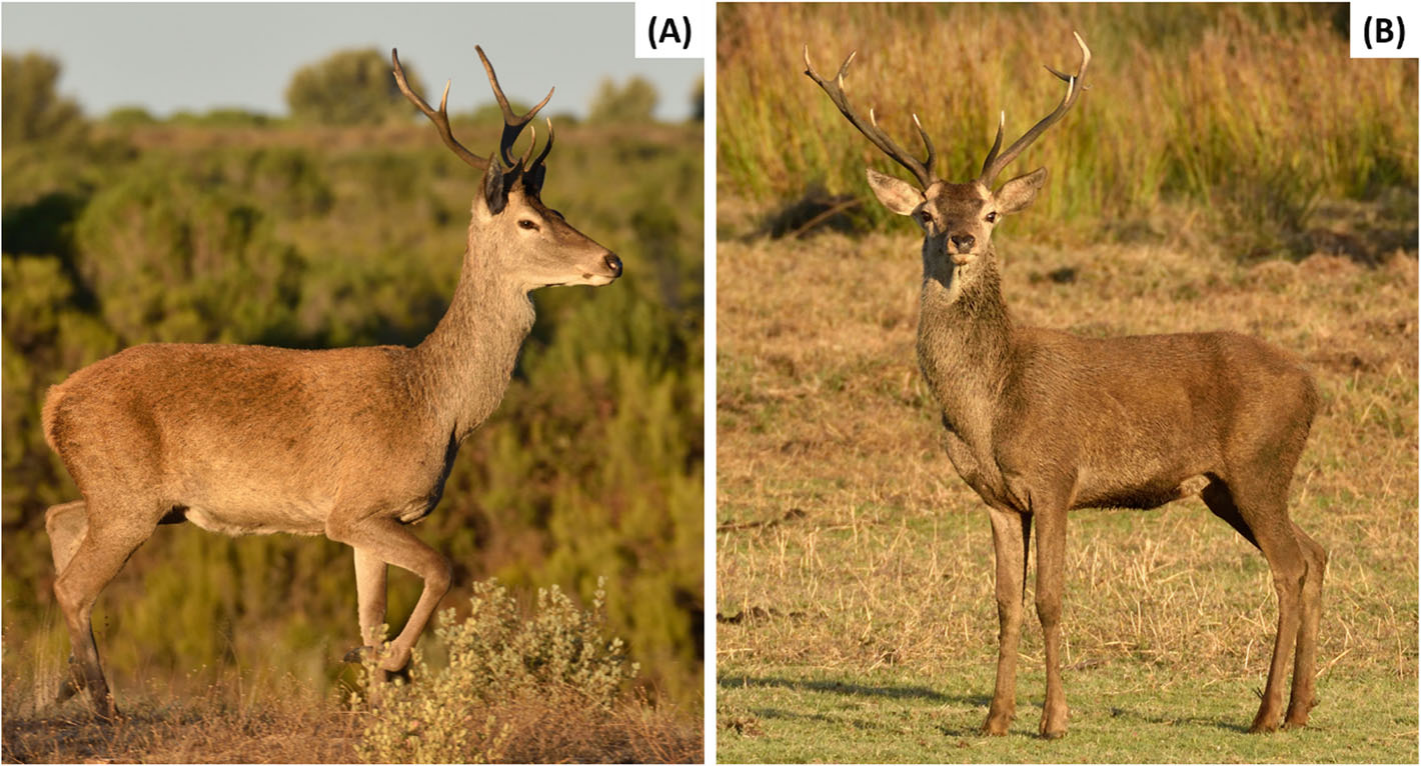
Ventral patch expression in two male Iberian red deer (*Cervus elaphus hispanicus*) (**A**) Low trait expression male (LTE) where the ventral surface shows hardly any dark coloration (**B**) High trait expression male (HTE) with a dark patch that occupies the entire ventral surface (Photograph by Jose Manuel Seoane)

Adult males were classified into two categories attending to the size of the dark ventral patch (trait expression): individuals with small or large dark ventral patches (low trait expression, LTE, vs. high trait expression, HTE; see [38]). We considered LTE males to those individuals in which the dark area was not observed or males with patches that only reached halfway down the ventral area (Fig. 2A). In contrast, HTE males showed the entire ventral area with dark coloration (Fig. 2B). Despite these two categories might appear arbitrary, they were supported by the clearly bimodal distribution of the dark ventral patch size in red deer, above and below 50 cm length [36–38] and this bimodality is maintained over time (see Additional file 1: Appendix S4).

### Behavioral observations

Behavioral data were collected during four rutting seasons for a total of 72 Iberian red deer males. Sampling time was restricted to the first hour after sunrise and to the three hours before sunset, coinciding with the main activity periods of this species [41]. To avoid bias, the observer was always the same person (EdP) throughout the study period, following the same data collection protocol. The methodologies used were either focal observations in situ or video recordings for later analysis, registering the start and end times of each focal observation.

In all cases, males were randomly selected to avoid bias in recording behaviors and they also were individually identified on the basis of morphological characteristics easily visible and identifiable by any observer (e.g., number of antler tines, a notch in the coat, singularities in antlers) [41, 60], so we were able to collect more than one observation per individual within a season. However, it was not possible to maintain this identification between sampled years, due to the annual renewal of the antlers. Consequently, we also explored the results for each year separately to prevent potential errors due to pseudo-replication (see below). A total of 227 focal observations corresponding to 72 individual males were observed over the four years. We decided to restrict the observation time of each individual to a maximum of 10 min to ensure to have time to collect behavioral data from an optimal large number of individuals and to obtain representative information from the same individuals at different times of the day throughout the mating season. Hence, the duration of individual observations ranged between 2 and 10 min and the mean observation time for each individual was 3.91 ± 0.41 min, with each individual being observed a mean of 3.15 times (range = 1–17 times). The total amount of observation time was 866.96 min and the distribution of observations among different individuals was not uniform.

Each male was categorized for trait expression as LTE or HTE (see above). During the behavioral observations, we could only count the number of antler tines of males as a morphological characteristic. However, we examined the relationship between the number of antler tines and age using morphometrical data of male Iberian red deer collected after hunting actions during sixteen consecutive years (see [82, 34] for methods). After using age determination described in Carranza et al. (2004) [82], we found a positive, curvilinear relationship between the number of antler tines and the age of males (Generalized Linear Model (GLZ) fitted to a Poisson distribution: *F*_1, 464.4_ = 749.83; *P* <  0.001).

We also recorded the males’ age category after visual inspection, classifying them as 2 years old or younger males, or sexually mature adult males including their body and antler developments [83]. Yearlings (1 year-old males) characterize by small body size and spike antlers without burr at their base. Young 2 years old males have a small body size but with burr and several tines in their antlers (two to five in each). Older males have bigger body sizes and many tines (typically more than five).

The number of females held by a male during each observation was noted as ‘harem size’. Adult individuals were classified into ‘territorial’ and ‘non-territorial’ males according to their defensive behavior previously defined by Carranza et al. (1990) [60, 84]. Different males’ sexual behaviors were recorded for each observation. Concretely, we identified and described seven male reproductive behaviors: roaring, antler rubbing, parallel walking, fighting, flehmen, female harassment, and female mounts (see Table 2). We noted the number of events where the focal individual performed each of these specific behaviors during an observation. We standardized frequencies of behaviors by observation time as a rate (behaviors/min). We also defined a new overall variable called ‘sexual activity’ as the summation of the rates of all these seven behaviors per each individual observation.

### Statistical analyses

We used data for all the four years to calculate mean rates of specific reproductive behaviors and overall mating activity for each male. In the following statistical analyses we did not include the observations of 2-year-old or younger males to avoid the error of taking into account the behavior of sexually immature individuals [85] when focussing on then effect of the ventral patch on the behavior of sexually mature males (see also Table 1 and Table 2).

We carried out Generalized Linear Mixed Models (GLMM) fitted to a binomial distribution, using the set of behavioral observations of adult males (*N* = 209) to show differences in mean values of each behavior between LTE and HTE males controlling by the individual as random factor (*N* = 60) (Table 2).

To explore the differences in overall mating activity between LTE and HTE adult males (*N* = 60), we carried out a Linear Mixed Model (LMM) fitted by restricted maximum likelihood (REML) where the dependent variable was ‘sexual activity’ and as covariables we included the number of antler tines, territoriality (territorial vs non-territorial) and harem size (LMM1). Ventral patch expression was included as a factor staged in two levels (LTE vs. HTE, see above). The sampled year and individual identity were included as random factors.

To check the effect of the dark ventral patch expression in the harem size of adult males (*N* = 60), we ran a Generalized Linear Mixed Model (GLMM) fitted to a Poisson distribution including as predictors the number of tines, sexual activity, and territoriality (territorial vs non-territorial) and trait expression (LTE vs. HTE). This model was checked for overdispersion (overdispersion parameter Ф < 1). As above, both sampled year and individual identity were included as random factors.

We carried out a Principal Component Analysis (PCA) with the seven described sexual behaviors to investigate which aspects of mating behavior were related to the dark ventral patch expression and to reduce the number of variables, losing the least possible amount of information. We included behavioral data of adult males (*N* = 60) along the four sampled years. We selected the principal components (PCs) with eigenvalues greater than 1 according to the Kaiser-Guttman criterion [86]. Loadings (i.e., the correlation between the variables and the components) represent how accurate is a component measuring an original variable [87]. Loadings under 0.3 were not considered when interpreting a factor.

Each of the four extracted principal component scores (PC1, PC2, PC3, and PC4) were used separately as a response variable in different LMMs (LMM2, LMM3, LMM4, and LMM5, respectively) to assess the relationship between the mating behavior and the dark ventral patch expression in adult males. The models included as fixed factors the trait expression (LTE vs. HTE) and the number of antler tines, as an indicator of males’ body condition. The sampled year and individual identity were included as random factors in all models.

In 2015 and 2018, no behavioral data for LTE individuals were recorded. To check that the obtained results were not biased by the unbalanced sample in these two years and due to the difficulty of identifying individual males, to avoid problems of pseudo-replication, we also ran the models only with years 2016 and 2017 (Additional file 1: Appendix S3) using the PC1 as a dependent variable. The results from both models were adjusted by multiple comparisons using the Bonferroni correction [88]. We also calculated the robust standard errors from the model using data from both years joined to control for sources of correlations in the data that we could not explicitly account. Due to reduced sample size, and to be conservative, we further confirmed model support by using estimates of significance between the selected versus the null model. These were obtained by parametric bootstrap procedures (‘PBmodcomp’) command from the R package *pbkrtest* following Halekoh and Højsgaard [89] of the final models using the PC1 as dependent variable and excluding as fixed factor the number of tines because of the non-significance of this covariable. Hence, we ran again a model, taking into account both 2016 and 2017 datasets and another similar model using only the year (2017) where we obtained the highest sample size. In addition to this, model parameters for the main effects in selected models are shown in Additional file 1: Appendix S4, and they were calculated from 1000 bootstrapped iterations derived with ‘bootMer’ (from the R package *lme4* [90]).

Models were checked for collinearity (all VIF < 3) and all quantitative explanatory variables were standardized in the models. For all statistical analyses a significant level of *P* < 0.05 was established and tests were carried out in R v.2.14.0 (R Foundation for Statistical Computing, Vienna, Austria), using *ade4* package for the PCA [91] and *lme4* package also for the LMMs and the GLMM [92].

## Supplementary Information

**Additional file 1: Appendix S1.** Frequencies (behavior rate per minute) of male Iberian red deer (*Cervus elaphus hispanicus*) mating behaviors displayed during 2016 and 2017 rutting seasons (*N* = 173). Table shows mean and standard deviations of frequencies, as well as Generalized Mixed Models results in which the frequency of each behavior was included as the dependent variable, the level of trait expressions (LTE vs HTE) as fixed factor, and individual (*N* = 173) as random factor. Each line in the GLMMs columns shows the mean differences and standard errors (SE) between trait expression, the Z statistic and significance level. The number of times a behaviour was recorded is shown as Obs (observed). **Appendix S2.** Results of LMM6 and LMM7 for the effect of ventral patch expression (LTE vs. HTE) of male Iberian red deer (*Cervus elaphus hispanicus*) on a Principal Component (PC1) representing reproductive activity, while controlling for the number of antler tines. The data set was split into the two sampled years. Results derived from the behavioral observations took in (**A**) 2016 (*N* = 8 males) and (**B**) 2017 (*N* = 32 males). Reference levels for factors are shown in brackets. Significant effects in bold (*p*-value = 0.05). Table also shows variance and standard errors (SE) of random effects (individual and year), as well as the residual variance of the model. **Appendix S3.** Parameter estimates and 95% confidence intervals of fixed effects from linear mixed models (LMM8 and LMM9; REML) testing the effects of the dark ventral patch expression (LTE vs. HTE) on the PC1 including the two sampled years where there were data of both adult LTE and HTE males (2016 and 2017; **A**) and the subset of 2017 (**B**) of male Iberian red deer (*Cervus elaphus hispanicus*). Estimate (± SE) = direction and magnitude of effect ± standard error; 95% C.I. = 95% Confidence interval from parametric bootstrapping (*n* = 1000); t-value = t-Student value with associated p-value. Random effects estimates are variance explained by random effects ± standard error. Reference levels for factors are shown in brackets. Significant terms (*p* < 0.05) are in bold. **Appendix S4.** Frequency histograms of the dark ventral patch size in male Iberian red deer showing the bimodality of trait expression of full hunting season collected individuals (**A**), October collected individuals (**B**), November and December collected individuals (**C**) and January and February individuals (**D**). Grey shading, low trait expression males (LTE, patch size between 0 and 50 cm); black shading, high trait expression males (HTE, patch size 50 cm and above). The mean trait size of each group is indicated by the dashed lines. Ventral patch measurements were collected 15 years after hunting activities in the southwestern Iberian Peninsula.

## Data Availability

Dataset used in this study is available at 10.6084/m9.figshare.12220802.v3.

## Abbreviations

LTE: low trait expression
HTE: high trait expression

## References

[1] Bradbury JW, Vehrencamp SL (1998). Principles of animal communication.

[2] Enquist M, Ghirlanda S, Hurd PL, Westneat D, Fox C (2010). Signalling evolutionary behavioural ecology.

[3] Andersson MB (1994). Sexual selection.

[4] Darwin C (1871). The descent of man and selection in relation to sex.

[5] Maynard Smith J (1991). Theories of sexual selection. Trends Ecol Evol.

[6] Johnstone RA. The evolution of animal signals. In: Behavioural ecology: an evolutionary approach. Blackwell Oxford. 1997. p. 155–78.

[7] Zahavi A (1997). The handicap principle: a missing piece of the Darwin’s puzzle.

[8] Maynard Smith J, Harper D (2003). Animal signals.

[9] Mougeot F, Irvine JR, Seivwright L, Redpath SM, Piertney S (2004). Testosterone, immunocompetence, and honest sexual signaling in male red grouse. Behav Ecol.

[10] Bortolotti GR, Blas J, Negro JJ, Tella JL (2006). A complex plumage pattern as an honest signal. Anim Behav.

[11] Velando A, Beamonte-Barrientos R, Torres R (2006) Pigment-based skin colour in the blue-footed booby: an honest signal of current condition used by females to adjust reproductive investment. Oecologia 149:135–142. 10.1007/s00442-006-0457-5 10.1007/s00442-006-0457-5 16821015

[12] Galeotti P, Sacchi R, Pellitteri-Rosa D, Fasola M (2011). The yellow cheek-patches of the Hermann's tortoise (Reptilia *Chelonia*): sexual dimorphism and relationship with body condition. Ital J Zool.

[13] Ibáñez A, Polo-Cavia N, López P, Martín J (2014). Honest sexual signaling in turtles: experimental evidence of a trade-off between immune response and coloration in red-eared sliders *Trachemys scripta elegans*. The Sci Nat.

[14] Rodríguez-Ruiz G, Ortega J, Cuervo JJ, López P, Salvador A, Martín J (2020). Male rock lizards may compensate reproductive costs of an immune challenge affecting sexual signals. Behav Ecol.

[15] Senar JC, Camerino M, Copete JL, Metcalfe NB (1993). Variation in black bib of the Eurasian siskin (*Carduelis spinus*) and its role as a reliable badge of dominance. Auk.

[16] Møller AP (1987). Variation in badge size in male house sparrows *Passer domesticus*: evidence for status signalling. Anim Behav.

[17] Liker A, Barta Z (2001). Male badge size predicts dominance against females in house sparrows. Condor.

[18] McGraw KJ, Dale J, MacKillop EA (2003) Social environment during molt and the expression of melanin-based plumage pigmentation in male house sparrows (*Passer domesticus*). Behav Ecol Sociobiol 53:116–122. 10.1007/s00265-002-0558-z

[19] Briffa M, Sneddon LU (2010) Contest behaviour. Evolutionary behavioral ecology. (ed) Westneat D and Fox C pp 246–265. Oxford: Oxford University press.

[20] Maynard Smith J, Parker GA (1976). The logic of asymmetric contests. Anim Behav.

[21] Tibbetts EA, Dale J (2004). A socially enforced signal of quality in a paper wasp. Nature.

[22] Tibbetts EA, Mullen SP, Dale J (2017). Signal function drives phenotypic and genetic diversity: the effects of signaling individual identity, quality or behavioural strategy. Philos Trans R Soc B.

[23] Tomkins JL, Hazel W (2007). The status of the conditional evolutionarily stable strategy. Trends Ecol Evol.

[24] Clutton-Brock TH, Albon SD (1979). The roaring of red deer and the evolution of honest advertisement. Behaviour.

[25] McComb KE (1987). Roaring by red deer stags advances the date of oestrus in hinds. Nature.

[26] McComb KE (1991). Female choice for high roaring in red deer *Cervus elaphus*. Anim Behav.

[27] Reby D, Hewison M, Izquierdo M, Pepin D (2001). Red deer (*Cervus elaphus*) hinds discriminate between the roars of their current harem-holder stag and those of neighbouring stags. Ethology.

[28] Clutton-Brock TH, Guinness F, Albon SD (1982). Red deer behavior and ecology of two sexes.

[29] Malo AF, Roldán ERS, Garde J, Soler AJ, Gomendio M (2005). Antlers honestly advertise sperm production and quality. Proc Soc Roy B.

[30] Reby D, McComb K (2003). Anatomical constraints generate honesty: acoustic cues to age and weight in the roars of red deer stags. Anim Behav.

[31] Fitch WT, Reby D (2001). The descended larynx is not uniquely human. Proc R Soc B.

[32] Davies NB, Krebs JR, West SA. An introduction to behavioural ecology. Wiley; 2012.

[33] Martín J, Carranza J, López P, Alarcos S, Pérez-González J (2014). A new sexual signal in rutting male red deer: age related chemical scent constituents in the belly black spot. Mammal Bio.

[34] Galván I, Solano F, Zougagh M, Andrés F, Murtada K, Ríos A, et al. Unprecedented high catecholamine production causing hair pigmentation after urinary excretion in red deer. Cell Mol Life Sci. 2019;76(2):397–404. 10.1007/s00018-018-2962-1. 10.1007/s00018-018-2962-1 PMC11105493 30413834

[35] de la Peña E, Martín J, Carranza J (2019). The intensity of male-male competition may affect chemical scent constituents in the dark ventral patch of male Iberian red deer. PLoS One.

[36] de la Peña E, Martín J, Barja I, Pérez-Caballero R, Acosta I, Carranza J (2020) The immune challenge of mating effort: steroid hormone profile dark ventral patch and parasite burden in relation to intrasexual competition in male Iberian red deer. Integr Zool. 10.1111/1749-487712427 10.1111/1749-4877.12427 31912636

[37] de la Peña E, Martín J, Barja I, Carranza J (2020). Testosterone and the dark ventral patch of male red deer: the role of the social environment. The Sci Nat.

[38] Carranza J, de la Peña E, Mateos C, Pérez-González J, Alarcos S, Torres-Porras J, et al. The dark ventral patch: a bimodal flexible trait related to male competition in red deer. PLoS One. 2020;15(11):e0241374. 10.1371/journal.pone.0241374. 10.1371/journal.pone.0241374 PMC7644014 33151970

[39] Searcy WA, Nowicki S (2005). The evolution of animal communication: reliability and deception in signalling.

[40] Martín J, Forsman A (1999). Social costs and development of nuptial coloration in male *Psammodromus algirus* lizards: an experiment. Behav Ecol.

[41] Carranza J, Valencia J (1999). Red deer females collect on male clumps at mating areas. Behav Ecol.

[42] Charlton BD, Reby D, McComb K (2007). Female red deer prefer the roars of larger males. Biol Lett.

[43] Rohwer S, Rohwer FC (1978). Status signalling in Harris sparrows: experimental deceptions achieved. Anim Behav.

[44] Gassett JW, Wiesler DP, Baker AG, Osborn DA, Miller KV, Marchinton RL, et al. Volatile compounds from interdigital gland of male white-tailed deer (*Odocoileus virginianus*). J Chem Ecol. 1996;22(9):1689–96. 10.1007/BF02272407. 10.1007/BF02272407 24226480

[45] Miller KV, Jemiolo B, Gassett JW, Jelinek I, Wiesler D, Novotny M (1998). Putative chemical signals from white-tailed deer (*Odocoileus virginianus*): social and seasonal effects on urinary volatile excretion in males. J Chem Ecol.

[46] Müller-Schwarze D (1971). Pheromones in black-tailed deer (*Odocoileus hemionus columbianus*). Anim Behav.

[47] Briffa M (2014). What determines the duration of war? Insights from assessment strategies in animal contests. PLoS One.

[48] Miyai CA, Sanches FHC, Costa TM, Colpo KD, Volpato GL, Barreto RE (2011). The correlation between subordinate fisheye colour and received attacks: a negative social feedback mechanism for the reduction of aggression during the formation of dominance hierarchies. Zoology.

[49] Todd PA, Wang WY, Huang H, Belle CC, Lim ML, Yeo DC (2011) The function of colourful facial bands in mangrove crab (*Perisesarma*) communication. J Exp Mar Bio Ecol 40:26–33. 10.1016/jjembe201107013

[50] Ewald PW, Rohwer S (1982). Effects of supplemental feeding on timing of breeding clutch-size and polygyny in red-winged blackbirds *Agelaius phoeniceus*. J Anim Ecol.

[51] Whitfield DP (1987). Plumage variability status signalling and individual recognition in avian flocks. Trends Ecol Evol.

[52] Pryke SR, Lawes MJ, Andersson S (2001). Agonistic carotenoid signalling in male red-collared widowbirds: aggression related to the colour signal of both the territory owner and model intruder. Anim Behav.

[53] Olsson M (1994). Rival recognition affects male contest behavior in sand lizards (*Lacerta agilis*). Behav Ecol Sociobiol.

[54] Hamilton DG, Whiting MJ, Pryke SR (2013). Fiery frills: carotenoid-based coloration predicts contest success in frillneck lizards. Behav Ecol.

[55] Simon VB (2011). Communication signal rates predict interaction outcome in the Brown anole lizard *Anolis sagrei*. Copeia.

[56] Ábalos J, Pérez I de Lanuza G, Carazo P, Font E (2016). The role of male colouration in the outcome of staged contests in the European common wall lizard (*Podarcis muralis*). Behaviour.

[57] Gerald MS (2001). Primate color predicts social status and aggressive outcome. Anim Behav.

[58] Caro T (2005) The adaptative significance of coloration in mammals. BioScience 551:125–136. 10.1641/0006-3568

[59] West PM, Packer C (2002). Sexual selection temperature and the lion’s mane. Science.

[60] Carranza J, Álvarez F, Redondo T (1990) Territoriality as a mating strategy in red deer. Anim Behav 40:79–88. 10.1016/S0003-3472(05)80667-0

[61] Estes RD (1972) The role of the vomeronasal organ in mammalian reproduction. Mammalia 36:315–341. 10.1515/mamm1972363315

[62] Müller-Schwarze D, Ritter F (1979). Flehmen in the context of mammalian urine communication. Chemical ecology: odour communication in animals.

[63] Spinage CA (1969) Naturalistic observations on the reproductive and maternal behaviour of the Uganda Defassa waterbuck (*Kobus defassa ugandae Neumann*) Z. Tierpsychol. 26:39–47. 10.1111/j1439-03101969tb01936x

[64] Ladewig J, Price EO, Hart BL (1980). Flehmen in male goats: role in sexual behaviour. Behav Neural Biol.

[65] Gaughwin MD (1979). The occurrence of flehmen in a marsupial - the hairy-nosed wombat (*Lasiorhinus latifrons*). Anim Behav.

[66] Henderson J, Altieri R, Müller-Schwarze D (1980). The annual cycle of flehmen in black-tailed deer (*Odocoileus hemionus columbianus*). J Chem Ecol.

[67] Wade MJ, Shuster SM (2004). Sexual selection: harem size and the variance in male reproductive success. Am Nat.

[68] Geist V, Thomas JW, Toweill DE (1982). Adaptive behavioural strategies. Elk of North America: ecology and management.

[69] Pemberton JM, Albon SD, Dover LE (1992). Behavioural estimates of male mating success tested by DNA fingerprinting in a polygynous mammal. Behav Ecol.

[70] Whiting MJ, Nagy KA, Bateman PW, Fox SF, Kelly Mccoy J, Baird TA (2003). Evolution and maintenance of social status signalling badges: experimental manipulations in lizards. lizard social behaviour.

[71] Senar JC (2006) Color displays as intrasexual signals of aggression and dominance. In bird coloration 2: function and evolution. (ed) Hill G and Mcgraw K. Harvard University press Cambridge pp 87–136.

[72] Tibbetts EA, Lindsay R (2008). Visual signals of status and rival assessment in *Polistes dominulus* paper wasps. Biol Lett.

[73] Chaine AS, Shizuka D, Block TA, Zhang L, Lyon BE (2018). Manipulating badges of status only fools strangers. Ecol Lett.

[74] Carranza J (2009). Defining sexual selection as sex-dependent selection. Anim Behav.

[75] Allier C, González F, Ramírez L (1974). Mapa Ecológico/Ecological map.

[76] Rogers PM, Myers K (1980). Animal distributions landscape classification and wildlife management. Coto Doñana Spain J Appl Ecol.

[77] Braza F, Álvarez F (1987). Habitat use by red deer and fallow deer in Doñana National Park. Miscelanea Zool.

[78] Carranza J (1995). Female attraction by males versus sites in territorial rutting red deer. Anim Behav.

[79] Carranza J, Fernández-Lario P, Gomendio M (1996) Correlates of territoriality in rutting red deer. Ethology 102:793–805. 10.1016/S0003-3472(05)80667-0

[80] Sánchez-Prieto CB, Carranza J, Pérez-González J, Alarcos S, Mateos C (2010). Effects of small barriers on habitat use by red deer: implications for conservation practices. J Nat Conserv.

[81] Passilongo D, Reby D, Carranza J, Apollonio M (2013) Roaring high and low: composition and possible functions of the Iberian stag's vocal repertoire PLoS One 8(5):e63841. 10.1371/journalpone0063841 10.1371/journal.pone.0063841 PMC3648515 23667678

[82] Carranza J, Alarcos S, Sánchez-Prieto C, Valencia J, Mateos C (2004). Disposable-soma senescence mediated by sexual selection in an ungulate. Nature.

[83] Pérez-González J, Carranza J (2011) Female aggregation interacts with population structure to influence the degree of polygyny in red deer. Anim Behav 82:957–970. 10.1016/janbehav201107023, 5, DOI: 10.1016/j.anbehav.2011.07.023

[84] Millán M, Carranza J, Pérez-González J, Valencia J, Torres-Porras J, Seoane J, et al. Rainfall decrease and red deer rutting behaviour: weaker and delayed rutting activity though higher opportunity for sexual selection. PLoS One. 2021;16(1):e0244802. 10.1371/journal.pone.0244802. 10.1371/journal.pone.0244802 PMC7817023 33471796

[85] Clutton-Brock TH, Rose KE, Guinness FE (1997) Density-related changes in sexual selection in red deer. Proc R Soc B 264:1509e1516. 10.1098/rspb19970209 10.1098/rspb.1997.0209 PMC1688709 9364790

[86] Kaiser HF (1960). The application of electronic computers to factor analysis. Educ Psych Meas.

[87] Comrey AL, Lee HB (1992) A first course in factor analysis second ed Lawrence Erlbaum. Associates Inc Publishers: Hillsdale. 10.4324/9781315827506

[88] Holm S. A simple sequentially rejective multiple test procedure. Scand J Statisc. 1979:65–70.

[89] Halekoh U, Højsgaard S (2014) A Kenward-Roger approximation and parametric bootstrap methods for tests in linear mixed models—the R package pbkrtest. J Stat Soft 59:1–32. 10.18637/jssv059i09

[90] Bates D, Mächler M, Bolker B, Walker S (2015) Fitting linear mixed-effects models using lme4. J Stat Soft 61:1–48. 10.18637/jssv067i01

[91] Dray S, Dufour AB (2007) The ade4 package: implementing the duality diagram for ecologists. J Stat Soft 22:1–20. 10.18637/jssv022i04

[92] Bates D, Mächler M, Bolker B, Walker S (2014). Fitting linear mixed-effects models using lme4. arXiv preprint arXiv:1406.5823.

