# Supplementary material for: The dark-ventral-patch of male red deer, a sexual signal that conveys the degree of involvement in rutting behavior
Source: BMC Zool. 2021 May 28;6:18. doi: 10.1186/s40850-021-00083-9 (PMC10127101; doi:10.1186/s40850-021-00083-9)
Supplement: Supplementary file 1 — Additional file 1: Appendix S1. Frequencies (behavior rate per minute) of male Iberian red deer (Cervus elaphus hispanicus) mating behaviors displayed during 2016 and 2017 rutting seasons (N = 173). Table shows mean and standard deviations of frequencies, as well as Generalized Mixed Models results in which the frequency of each behavior was included as the dependent variable, the level of trait expressions (LTE vs HTE) as fixed factor, and individual (N = 173) as random factor. Each line in the GLMMs columns shows the mean differences and standard errors (SE) between trait expression, the Z statistic and significance level. The number of times a behaviour was recorded is shown as Obs (observed). Appendix S2. Results of LMM6 and LMM7 for the effect of ventral patch expression (LTE vs. HTE) of male Iberian red deer (Cervus elaphus hispanicus) on a Principal Component (PC1) representing reproductive activity, while controlling for the number of antler tines. The data set was split into the two sampled years. Results derived from the behavioral observations took in (A) 2016 (N = 8 males) and (B) 2017 (N = 32 males). Reference levels for factors are shown in brackets. Significant effects in bold (p-value = 0.05). Table also shows variance and standard errors (SE) of random effects (individual and year), as well as the residual variance of the model. Appendix S3. Parameter estimates and 95% confidence intervals of fixed effects from linear mixed models (LMM8 and LMM9; REML) testing the effects of the dark ventral patch expression (LTE vs. HTE) on the PC1 including the two sampled years where there were data of both adult LTE and HTE males (2016 and 2017; A) and the subset of 2017 (B) of male Iberian red deer (Cervus elaphus hispanicus). Estimate (± SE) = direction and magnitude of effect ± standard error; 95% C.I. = 95% Confidence interval from parametric bootstrapping (n = 1000); t-value = t-Student value with associated p-value. Random effects estimates are variance ex [file 40850_2021_83_MOESM1_ESM.docx]

**The dark-ventral-patch of male red deer, a sexual signal that conveys the degree of involvement in rutting behavior**

Eva de la Peña^1^, Javier Pérez-González^1,2^, José Martín^3^, Giovanni Vedel^1^, Juan Carranza^1^

^1^Wildlife Research Unit (UIRCP), University of Córdoba, 14071 Córdoba, Spain.

^2^Biology & Ethology, University of Extremadura, 10071 Cáceres, Spain.

^3^Department of Evolutionary Ecology, Museo Nacional de Ciencias Naturales (MNCN-CSIC), 28006, Madrid, Spain.

Corresponding author: Eva de la Peña ([evadelapenha@gmail.com](mailto:evadelapenha@gmail.com); 0034690393915)

**Supplementary Information**

**Appendix S1**. Frequencies (behavior rate per minute) of male Iberian red deer (*Cervus elaphus hispanicus*) mating behaviors displayed during 2016 and 2017 rutting seasons (N = 173). Table shows mean and standard deviations of frequencies, as well as Generalized Mixed Models results in which the frequency of each behavior was included as the dependent variable, the level of trait expressions (LTE vs HTE) as fixed factor, and individual (N = 173) as random factor. Each line in the GLMMs columns shows the mean differences and standard errors (SE) between trait expression, the Z statistic and significance level. The number of times a behaviour was recorded is shown as Obs (observed).

| **Mating behaviour** | **Mean frequency ± SD** | | **GLMMs** | | |
| --- | --- | --- | --- | --- | --- |
|  | **LTE** | **HTE** | Estimate ± SE | *Z* | *P* |
| Roaring (Obs = 146) | 0.47 ± 0.74 | 2.17 ± 1.59 | -3.411 0.986 | -3.459 | < 0.001 |
| Flehmen (Obs = 38) | 0.01 ± 0.03 | 0.16 ± 0.47 | -7.528 ± 8.722 | -0.863 | 0.388 |
| Antler rubbing (Obs =50) | 0.04 ± 0.18 | 0.17 ± 0.43 | -0.182 ± 0.497 | -0.365 | 0.715 |
| Parallel walk (Obs =13) | 0.01 ± 0.04 | 0.02 ± 0.07 | -0.601 ± 1.131 | -0.532 | 0.595 |
| Fight (Obs = 4) | 0.01 ± 0.07 | 0.0 ± 0.04 | 0.107 ± 0.227 | 0.470 | 0.638 |
| Female harassment (Obs = 76) | 0.09 ± 0.30 | 0.34 ± 0.52 | -7.380 ± 5.943 | -1.242 | 0.214 |
| Mount female (Obs = 1) | 0.00 ± 0.00 | 0.001 ± 0.02 | -3.370 ± 236.067 | -0.014 | 0.989 |
| Sexual activity (Obs = 173) | 0.63 ± 0.90 | 2.88 ± 2.06 | -3.667 ± 0.950 | -3.859 | < 0.001 |

|  |  |
| --- | --- |
|  |  |
|  |  |
|  |  |
|  |  |
|  |  |
|  |  |
|  |  |
|  |  |

**Appendix S2**. Results of LMM6 and LMM7 for the effect of ventral patch expression (LTE vs. HTE) of male Iberian red deer (*Cervus elaphus hispanicus*) on a Principal Component (PC1) representing reproductive activity, while controlling for the number of antler tines. The data set was split into the two sampled years. Results derived from the behavioral observations took in (A) 2016 (N = 8 males) and (B) 2017 (N = 32 males). Reference levels for factors are shown in brackets. Significant effects in bold (*p*-value = 0.05). Table also shows variance and standard errors (SE) of random effects (individual and year), as well as the residual variance of the model.

| **(A) Year: 2016** | Estimate (± SE) | df | *t* | *P* |
| --- | --- | --- | --- | --- |
| Fixed factors | | | | |
| Intercept | 0.6563 ± 1.605 | 7.518 | 0.409 | 0.711 |
| Trait expression (HTE) | - 0.077 ± 0.595 | 8.151 | - 1.294 | 0.241 |
| Antler tines | - 0.041 ± 0.098 | 6.936 | - 0.423 | 0.706 |
| Random factors:  *Individual*: variance ± SE = 0.115 ± 0.339; Residual = 0.384 ± 0.619 | | | | |
| **(B) Year: 2017** | Estimate (± SE) | df | *t* | *P* |
| Fixed factors | | | | |
| Intercept | 0.391 ± 0.685 | 22.356 | 0.571 | 0.599 |
| Trait expression (HTE) | - 1.146 ± 0.391 | 126.685 | - 2.928 | **0.004** |
| Antler tines | - 0.009 ± 0.054 | 21.419 | - 0.184 | 0.866 |
| Random factors:  *Individual*: variance ± SE = 0.000 ± 0.000; Residual = 1.656 ± 1.287 | | | | |

**Appendix S3.** Parameter estimates and 95% confidence intervals of fixed effects from linear mixed models (LMM8 and LMM9; REML) testing the effects of the dark ventral patch expression (LTE vs. HTE) on the PC1 including the two sampled years where there were data of both adult LTE and HTE males (2016 and 2017; A) and the subset of 2017 (B) of male Iberian red deer (*Cervus elaphus hispanicus*). Estimate (± SE) = direction and magnitude of effect ± standard error; 95% C.I. = 95% Confidence interval from parametric bootstrapping (n = 1000); t-value = t-Student value with associated *p*-value. Random effects estimates are variance explained by random effects ± standard error. Reference levels for factors are shown in brackets. Significant terms (p < 0.05) are in bold.

| **(A)** | Estimate (± SE) | 95% CI | *t* | *P* |
| --- | --- | --- | --- | --- |
| Fixed factors | | | | |
| Intercept | - 0.238 ± 0.103 | - 0.017 to 0.484 | 1.994 | 0.783 |
| Trait expression (HTE) | - 1.069 ± 0.334 | - 1.731 to - 0.424 | - 3.261 | **0.001** |
| Random factors:  *Individual*: variance ± SE = 0.000 ± 0.000; *Year* = 0.000 ± 0.000; Residual = 0.578 ± 0.761 | | | | |
| **(B)** | Estimate (± SE) | 95% CI | *t* | *P* |
| Fixed factors | | | | |
| Intercept | 0.267 ± 0.115 | 0.405 to 0.501 | 2.258 | **0.040** |
| Trait expression (HTE) | - 1.127 ± 0.388 | - 1.878 to - 0.342 | - 2.944 | **0.003** |
| Random factors:  *Individual*: variance ± SE = 0.000 ± 0.000; Residual = 1.645 ± 1.282 | | | | |

**
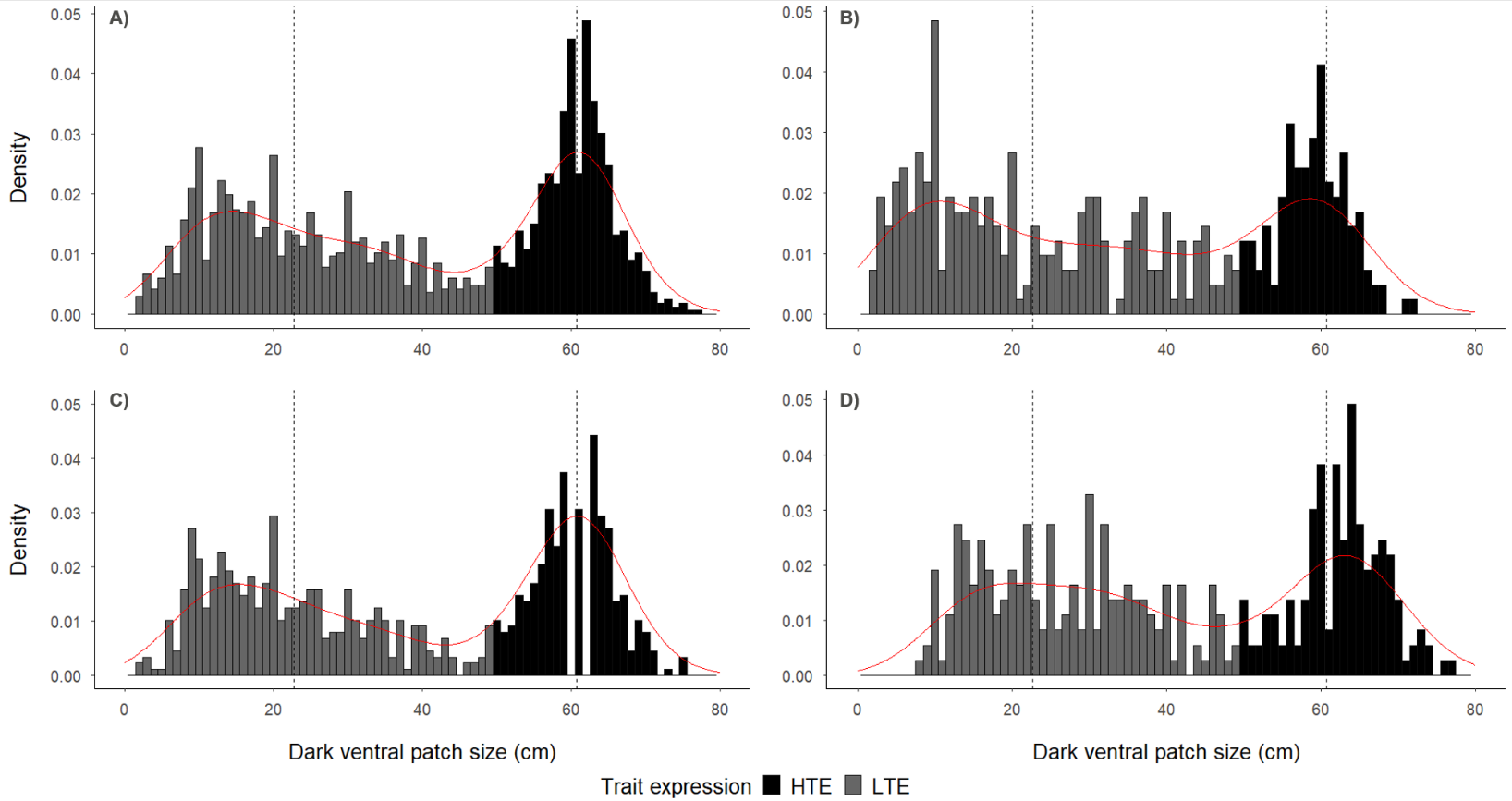
Appendix S4**. Frequency histograms of the dark ventral patch size in male Iberian red deer showing the bimodality of trait expression of full hunting season collected individuals (A), October collected individuals (B), November and December collected individuals (C) and January and February individuals (D). Grey shading, low trait expression males (LTE, patch size between 0 and 50 cm); black shading, high trait expression males (HTE, patch size 50 cm and above). The mean trait size of each group is indicated by the dashed lines. Ventral patch measurements were collected 15 years after hunting activities in the southwestern Iberian Peninsula.
